# In Vitro Bactericidal Activity of a Neomycin-Polymyxin B-Nystatin Combination Compared to Metronidazole and Clindamycin Against the Main Bacteria Involved in Bacterial Vaginosis and Aerobic Vaginitis

**DOI:** 10.3390/ph18030340

**Published:** 2025-02-27

**Authors:** Catherine Feuillolay, Sylvie Salvatico, Julie Escola, Barbara Quioc-Salomon, Frédéric Carrois, Christine Roques

**Affiliations:** 1 ACM Pharma Fonderephar, 35 Chemin des Maraîchers, 31062 Toulouse, France; catherine.feuillolay@fonderephar.com (C.F.);; 2 Laboratoire Innotech International, Groupe Innothera, 22 Avenue Aristide Briand, 94110 Arcueil, Francebarbara.quioc-salomon@innothera.com (B.Q.-S.); frederic.carrois@innothera.com (F.C.); 3 Laboratoire de Génie Chimique UMR 5503 (CNRS, INPT, UPS), Université de Toulouse, 35 Chemin des Maraîchers, 31062 Toulouse Cedex 9, France

**Keywords:** aerobic vaginitis, antibiotic combination, clindamycin, metronidazole, neomycin, nystatin, polymyxin B, vaginal infection, vaginosis

## Abstract

**Background/Objectives:** Aerobic vaginitis (AV) and bacterial vaginosis (BV) are vaginal infections requiring the fast elimination of pathogens. The frequent confusion of these infections may justify the use of a rapidly acting broad-spectrum antibiotic treatment. **Methods:** This study investigated the bactericidal kinetics of the neomycin-polymyxin B-nystatin (NPN) combination compared to those of two reference antibiotics (clindamycin and metronidazole) against 22 bacteria commonly implicated in AV and BV. **Results:** NPN exhibited bactericidal activity against the aerobic Gram-positive bacteria, with particularly high bactericidal activity being observed against streptococci, *S. aureus*, and *C. amycolatum* after 1 h at low dilutions and after 4 h for all dilutions. Enterococci were less sensitive to NPN. Clindamycin demonstrated poor rapid bactericidal activity against all Gram-positive bacteria tested. NPN manifested high bactericidal activity against all aerobic Gram-negative bacteria tested, whereas clindamycin showed bactericidal activity only after 4 h at a 1/2 dilution. With respect to the four anaerobic strains tested, NPN demonstrated high bactericidal activity at all tested dilutions with concentration-dependent effects. Metronidazole exhibited lower or no rapid bactericidal activity. **Conclusions:** These results suggest that NPN has very fast bactericidal action against the main bacteria involved in AV and BV compared to clindamycin and metronidazole, highlighting its potential in managing bacterial vaginal infections.

## 1. Introduction

Vaginal infections are the most common reason for gynecological consultations [1]. They may contribute to the progression of various diseases and increase the risk of post-delivery infections, as well as resulting in substantial discomfort and poor self-esteem, leading to impaired quality of life [2].

Abnormal vaginal discharges may be caused by a wide range of well-described infectious agents and diseases [3], ranging from vulvovaginal candidiasis to bacterial vaginosis (BV) [4,5], aerobic vaginitis (AV) [6], or mixed vaginitis, as well as sexually transmitted infections including *Neisseria gonorrhoeae*, *Chlamydia trachomatis*, *Ureaplasma*, and the parasite *Trichomonas vaginalis*. BV is considered the main cause of vaginal infections, with the prevalence in the general population ranging from 23% to 29%, depending on the location [7]. BV constitutes a state of dysbiosis in the vaginal microbiota, in which typically dominant *Lactobacillus* species are displaced by an array of BV-associated species [8,9]. The etiology of this dysbiosis and *Lactobacillus* displacement remains unknown, although some authors believe that certain species of *Gardnerella* play a necessary but not unique role in initiating this process [10,11,12]. At the same time, microbial profiles evolve, and anaerobic species have been detected with a high prevalence in BV-positive women. The conclusions of individual studies vary as to which bacteria are considered important in the diagnosis or prognosis of BV, but the most frequently involved species are *Atopobium* sp., *Dialister* sp., *Prevotella* sp., *Mobiluncus* sp., *Fusobacterium* sp., *Peptinophilus* sp., and *Corynebacterium* sp. [13,14,15,16,17,18,19,20,21]. BV is therefore considered typically polymicrobial, characterized by the presence of *G. vaginalis* associated with mainly anaerobic bacteria [21,22], an increased vaginal pH (≥4.5), the presence of clue cells, and a fishy odor [23]. AV is less prevalent than BV, with prevalences ranging from 7 to 12% [24]. AV, despite also being defined by disruption to *Lactobacillus* dominance, differs from BV by involving more extreme inflammatory changes and the presence of mainly aerobic enteric bacteria, including group B streptococci (such as *S. agalactiae*), *Enterococcus faecalis*, *Escherichia coli*, and *Staphylococcus aureus* [24,25,26,27]. BV and AV pose significant risks, including obstetrical complications and an increased risk of sexually transmitted infection acquisition (e.g., human papilloma virus, human immunodeficiency virus, *Trichomonas vaginalis*, and *Chlamydia trachomatis*). Therefore, it is crucial to treat these infections promptly [28].

The aim of BV and AV treatment is to restore the balance of the vaginal flora and thereby limit the excessive growth of harmful microorganisms. Current international guidelines recommend the use of antibiotics, in particular, metronidazole or clindamycin, topically in the first instance and then, depending on disease evolution, systemically [28,29,30,31,32,33,34]. Local treatments are generally preferred as a first-line treatment because oral options can result in systemic side effects such as headache and gastro-intestinal disorders. In contrast, vaginal applications can reach local concentrations up to 30 times higher, resulting in comparable or slightly improved cure rates with fewer side effects [28]. As clindamycin is active against both staphylococci and streptococci, as well as anaerobes, the International Union Against Sexually Transmitted Infections states that the best treatment for uncomplicated AV is currently vaginally administered clindamycin, whereas metronidazole if advocated for the treatment of persistent and recurrent BV [3,28,29]. However, these first-line treatments may have shortcomings, as metronidazole is associated with high levels of failure and recurrence in patients presenting BV [13,35,36,37,38], while clindamycin may not inhibit all species involved in AV [24]. Clinical cure rates ranging from 58% to 92% have been observed after one month of oral or local treatment [32], although recurrences or re-infections have been observed at rates exceeding 50% within the following year [35,39]. High recurrence rates after metronidazole treatment have been observed, reaching up to 80 percent [35,40,41,42]. Only a few recommendations exist regarding the treatment of AV, and they are mainly based on limited observational studies and expert opinions [28]. Even though several guidelines recommend using kanamycin or clindamycin, the best treatment has not yet been fully determined [24].

High rates of therapeutic failure and recurrences could be explained by inconsistencies in the diagnosis of BV and AV. AV shares several characteristics with BV, such as a diminished number or absence of lactobacilli, abnormal vaginal discharges, and an elevated vaginal pH [24], and can be misdiagnosed as BV. In addition, clinical findings are insufficient to distinguish between AV or BV alone and mixed infections [43]. Microscopy combined with adequate scoring systems remains largely underused in clinical practice [24], the specified diagnostic tools have some drawbacks, and, in general, diagnosis remains problematic and challenging [44].

Without an accurate diagnosis, BV and AV may be incorrectly identified, leading to inappropriate or incomplete treatment, increasing the risk of therapeutic failure and complications. These common diagnostic uncertainties and the frequency of mixed vaginitis underscore the importance of the consideration of broad-spectrum local treatment and polytherapy [45,46]. The neomycin-polymyxin B-nystatin (NPN) combination, an antibiotic/antifungal combination, has shown high clinical efficacy against various types of infectious vaginitis, including bacterial vaginitis (85.4%), bacterial vaginosis (96.4%), and mixed vaginitis (87.7%), with a fast reduction in symptoms [47]. While NPN contains the antifungal agent nystatin, the present study focused solely on its bactericidal properties. Indeed, the in vitro spectrum and kinetics of its bactericidal activity have not yet been fully elucidated or compared with those of alternative treatments.

The objective of the in vitro study reported here was to determine the kinetics of the bactericidal activity of the NNP combination against the most common bacteria involved in AV and in BV versus those of the corresponding two reference antibiotics considered as first-line therapy (metronidazole for BV and clindamycin for AV). The originality of this objective lay in the evaluation of bactericidal activity (i) for short contact times (1 h and 4 h), (ii) according to antibiotic dilution, with the aim of assessing the duration of bactericidal activity, and (iii) on a large panel of bacterial species implicated in BV and AV, respectively.

## 2. Results

### 2.1. Bactericidal Activity Against Bacteria Involved in Bacterial Vaginosis

**Figure 1:**
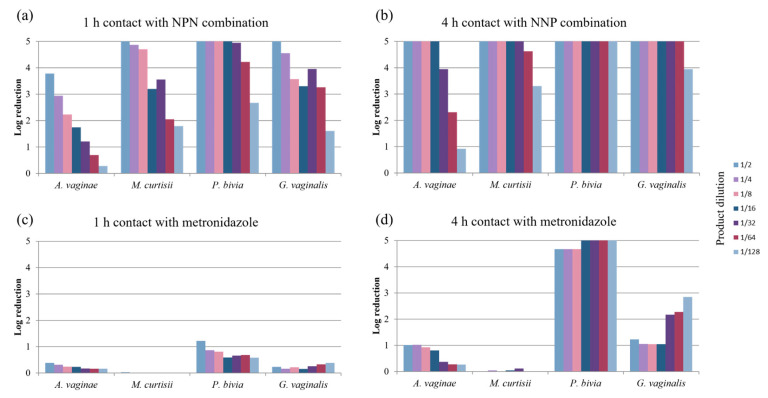
Log reductions in populations of the main bacteria implicated in BV after (**a**) 1 h contact with NPN combination, (**b**) 4 h contact with NPN combination, (**c**) 1 h contact with metronidazole, and (**d**) 4 h contact with metronidazole, in the presence of calf serum, according to dilution (from 1/2 to 1/128). The maximum reduction observed was ≥5 log.

The bactericidal activity of the NPN combination was tested against the main bacteria involved in bacterial vaginosis after 1 h (Figure 1a) and 4 h (Figure 1b) of contact, according to the dilution. The results are presented as log reductions in bacterial colony-forming unit (CFU) counts. Under the assay conditions employed, including the presence of calf serum to simulate the vaginal environment, a concentration-dependent effect was observed after 1 h of contact for *Atopobium vaginae*, with a log reduction ranging from 4 (1/2 dilution) to 1 (1/128 dilution). High log reductions (≥5) were also noted after 4 h of contact with dilutions up to 1/16. In particular, high log reductions (≥4) were similarly noted for *Mobiluncus curtisii* in each of the conditions tested with regard to the 1/2 and to 1/8 dilutions. Assays performed after 1 h of contact indicated a concentration-dependent effect with a log reduction ranging from 5 (1/2 dilution) to less than 2 (1/128 dilution). High log reductions were maintained for all the tested dilutions even after 4 h of contact. Log reductions ≥ 5 were also observed with respect to *Prevotella bivia* at both contact times up to the 1/16 dilutions. Assays performed after 1 h of contact indicated a concentration-dependent effect but, invariably, a significant reduction even at the 1/128 dilution (with an approximately 3 log reduction). High log reductions (≥5) were maintained for all the tested dilutions even after 4 h of contact. Finally, for *Gardnerella vaginalis*, assays performed after 1 h of contact indicated a concentration-dependent effect, with log reductions ranging from about 5 (for the 1/2 dilution) to about 2 (for the 1/128 dilution). High log reductions (≥5) were noted after 4 h of contact up to the 1/64 dilution.

Regarding metronidazole, no significant bactericidal activity was noted for a 1 h contact time (log reduction ≤ 1 log) against the four bacteria tested (Figure 1c), except for *P. bivia* at the dilution 1/2. For *P. bivia*, increasing the contact time to 4 h (Figure 1b,d) led to higher log reductions (4 to 5 log). With respect to *G. vaginalis*, increasing the contact time to 4 h (Figure 1b,d) led to a 1 log reduction for dilutions 1/2 to 1/16 and to log reductions of 2–3 for subsequent dilutions. This inverse dose effect is worth emphasizing, as it is possibly linked to an interference effect within the formulation. The bactericidal activity of metronidazole was close to zero for *A. vaginae* and *M. curtisii* after contact times of 1 h and 4 h.

In conclusion, with regard to the main bacteria implicated in BV, the NPN combination exhibited a very homogenous and high bactericidal activity against the four bacterial strains tested, increasing with the contact time from 1 h to 4 h. This bactericidal effect was maintained even at high dilutions and in the presence of calf serum. Metronidazole was characterized by poor or no bactericidal activity against *A. vaginae* and *M. curtisii*. With respect to *G. vaginalis*, only log reductions less than 3 were noted after a 4 h contact time. High log reductions were only observed for *P. bivia* after a 4 h contact time. These results are consistent with a better sensitivity to metronidazole in *P. bivia* and secondarily in *G. vaginalis* in terms of MICs (see Supplementary Data).

### 2.2. Bactericidal Activity Against Bacteria Involved in Aerobic Vaginitis

The bactericidal activity of the NPN combination and clindamycin was evaluated against Gram-positive and Gram-negative bacteria involved in aerobic vaginitis (AV).

#### 2.2.1. Gram-Positive Bacteria

**Figure 2:**
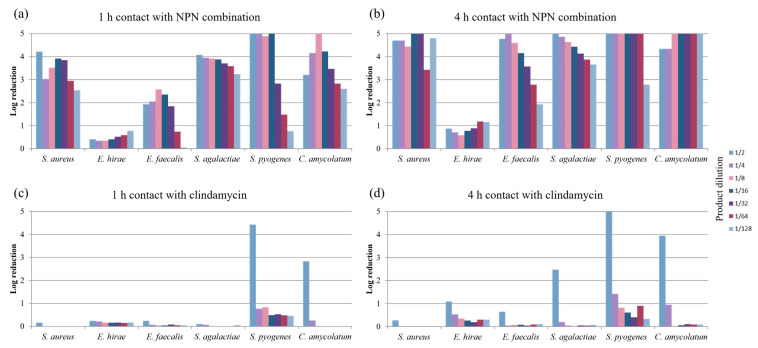
Log reductions in populations of the main Gram-positive bacteria implicated in AV after (**a**) 1 h contact with NPN combination, (**b**) 4 h contact with NPN combination, (**c**) 1 h contact with clindamycin, and (**d**) 4 h contact with clindamycin, in the presence of calf serum, according to dilution (from 1/2 to 1/128). The maximum reduction observed was ≥5 log.

Regarding Gram-positive bacteria, the bactericidal activity of the NNP combination differed according to the bacterial species. For streptococci (*S. agalactiae* and *S. pyogenes*), *S. aureus*, and *C. amycolatum*, log reductions exceeding 3 log were observed after only 1 h of contact for dilutions of 1/2 to 1/16 (Figure 2a). Increasing the contact time from 1 h to 4 h led to progressively greater log reductions in bacterial populations, reaching 4 to 5 log reductions at most dilutions tested (Figure 2b). Enterococci appeared to be the least sensitive to the NNP combination, especially *E. hirae*, against which the log reduction in the bacterial population reached only one log whatever the conditions in terms of contact time and dilution. In the case of *E. faecalis*, a contact time of 1 h led to a significant log reduction (above 1 log) for the dilutions of 1/2 to 1/16 (Figure 2a). With a 4 h contact time, log reductions increased to more than 4 log for the same dilutions (Figure 2b).

In contrast, clindamycin, considered bacteriostatic against Gram-positive bacteria, exhibited much lower bactericidal activities than the NPN combination with the short contact times tested (1 h, Figure 2c and 4 h, Figure 2d). Log reductions above 2 and 4 were observed only for the 1/2 dilution after 1 h of contact for *C. amycolatum* and *S. pyogenes*, respectively (Figure 2c). For the other strains tested, a contact period of 1 h with clindamycin dilutions gave rise to a very poor log reduction in bacterial populations (less than 1 log) (Figure 2c). Increasing the contact time to 4 h (Figure 2d) did not achieve significant log reductions at higher dilutions, with reductions of only 1 to <2.5 log being observed for *E. hirae* and *S. agalactiae*, respectively (Figure 2d). Surprisingly, *S. aureus* appeared to be the least sensitive to clindamycin under the assay conditions tested, despite an MIC value of 0.06 mg/L, underlining an essentially growth-inhibitory activity (Supplementary data).

These results demonstrated greater sensitivity, expressed as bactericidal activity, in *S. pyogenes* to both the antibiotic products evaluated (only for high concentrations in the case of clindamycin), in comparison to the other Gram-positive strains tested.

#### 2.2.2. Gram-Negative Bacteria

**Figure 3:**
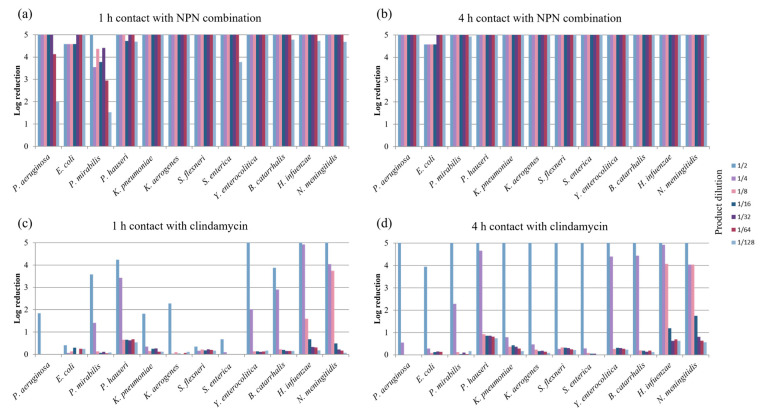
Log reductions in populations of the main Gram-negative bacteria implicated in AV after (**a**) 1 h contact with NPN combination, (**b**) 4 h contact with NPN combination, (**c**) 1 h contact with clindamycin, and (**d**) 4 h contact with clindamycin, in the presence of calf serum, according to dilution (from 1/2 to 1/128). The maximum reduction observed was ≥5 log.

Regarding Gram-negative bacteria, the NPN combination presented a high bactericidal activity overall against all the bacterial strains tested, including the Gram-negative bacilli *P. aeruginosa*, the Enterobacteriaceae *B. catarrhalis* and *H. influenza*, and the Gram-negative cocci *N. meningitidis* (Figure 3). The least sensitive strain was *P. mirabilis*, for which a log reduction ≥ 5 log was observed only for the 1/2 dilution after a contact time of 1 h, with log reductions decreasing progressively at increasing dilutions but remaining above 1 log at the 1/128 dilution (Figure 3a). With respect to the other bacterial strains, a decrease in log reduction after a contact time of 1 h was observed only for the dilutions from 1/64 to 1/128 (Figure 3a). After a 4 h contact time, log reductions reached values ≥ 5 log for all the Gram-negative strains tested (Figure 3b).

Regarding our objective of evaluating overall bactericidal activity after short contact times, in line with clinical antibiotic use, clindamycin did have a significant bactericidal effect on Gram-negative bacteria but only at the lowest dilutions tested (1/2 and 1/4) (Figure 3c,d). At higher dilutions, a dramatic decrease in bactericidal activity was observed. With regard to the Gram-negative strain tested, a significant log reduction after 1 h of contact with the dilutions 1/2 and 1/4 was observed for the Gram-negative cocci *N. meningitidis*, *H. influenza*, and *B. catarrhalis*. *P. aeruginosa* and Enterobactericeae appeared to be less sensitive (with the exception of *Y. enterocolitica*, *P. hauseri*, and *P. mirabilis*) (Figure 3c). After a 4 h contact time (Figure 3d), log reductions reached values of around 5 log (or above the detection limit) for all the Gram-negative strains tested but only for the 1/2 dilution. The 4 h bactericidal activity was partially maintained against the most sensitive bacteria defined after 1 h of contact for dilutions of 1/4 and 1/8.

The NPN combination also expressed a high bactericidal activity against the main bacteria implicated in AV, both Gram-negative and Gram-positive strains, including *S. aureus* and streptococci, with the exception of enterococci, at all dilutions tested (from 1/2 to 1/128) after short contact times (1 h and 4 h) and in the presence of calf serum. In contrast, clindamycin, considered an effective antibacterial agent against Gram-positive bacteria, showed poor bactericidal activity under our assay conditions. Increasing the contact time from 1 h to 4 h lead to significant log reductions only for dilutions beyond 1/4.

## 3. Discussion

This study demonstrates the broad-spectrum bactericidal activity of the NNP combination, effective on both aerobic and anaerobic and Gram-positive and Gram-negative bacteria. The broad-spectrum bactericidal activity of polymyxin B combined with neomycin may be partially explained by their consistent modes of action. Polymyxins are characterized by a net-positive charge and the presence of hydrophobic regions, permitting their interactions with LPS [48]. As a result of these electrostatic and hydrophobic interactions, the bacterial outer membrane is disrupted, leading to the “self-promoted” uptake of polymyxins [48,49], which is also relevant in other antimicrobial substances, including other antibiotics [49]. The bactericidal activity of Polymyxin B is known to involve reactive oxygen species production, inducing DNA, protein, and lipid damage [50]. Polymyxins are active against most members of the Enterobacteriaceae family but also against common non-fermentative Gram-negative bacteria such as *Pseudomonas aeruginosa* [51]. At the same time, Polymyxins are considered inefficient against *Neisseria* sp. and strict anaerobes. From a pharmacological point of view, Polymyxin B is administered as its sulfate salt, which leads to direct activity in the antimicrobial entity for topical uses [52].

Neomycin, as an aminoglycoside, binds to the 16S rRNA and inhibits protein synthesis with pleiotropic translational effects that decrease tRNA selection accuracy [53] and inhibit spontaneous and EFG-catalyzed translocation [54,55,56,57]. Neomycin shows broad-spectrum activity toward aerobic Gram-positive and Gram-negative bacteria. As highlighted in the Supplementary Data, the individual spectra of neomycin and polymyxin B often exhibit complementarity. While neomycin primarily targets some Gram-positive and Gram-negative species, polymyxin B is more effective against Gram-negative pathogens, including those resistant to neomycin. This study defined the activity spectrum of the combination, which also includes anaerobic strains. The positive interaction noted with different combined modes of action likely contributes to the rapid and potent bactericidal activity of the NNP combination. This broad spectrum and rapid bactericidal activity could lead to the faster relief of symptoms while targeting most of the bacteria involved in vaginal infections, including cases when etiologies are mixed. More clinical studies assessing its efficacy, as well as the impact of this broad spectrum on the recurrency of infections or on biofilms, are needed to better define its therapeutic role.

Although metronidazole is considered the gold standard for the treatment of BV, in our experiments, it was characterized by poor or no in vitro rapid bactericidal activity against *A. vaginae* and *M. curtisii* and limited rapid bactericidal activity against *G. vaginalis*. The high rate of recurrence noted after metronidazole treatment has been frequently linked to metronidazole resistance, as demonstrated in vitro [40,58,59,60,61,62,63] or to in vivo inactivation or sequestration by certain microorganisms [64], including when under biofilms [37,65,66,67,68,69]. Mollin et al. demonstrated that patients experiencing recurrence after transient remission had manifested an elevated abundance of bacterial species, such as *A. vaginae*, *G. vaginalis*, and *Aerococcus christensenii*, compared to patients in long-term remission [70]. The abundance of core bacterial species in refractory patients did not decrease after metronidazole treatment, suggesting resistance or tolerance, in contrast to the diminishing abundance of these same species in patients experiencing successive recurrence and remission patients. Recently, Li et al. demonstrated in vitro that clinical isolates of *G. vaginalis* showed a susceptibility rate of 76.67% and a resistance rate of 23.33% to clindamycin, with significantly less favorable values for metronidazole (susceptibility rate, 38.24%; resistance rate, 58.82%) [68].

The lack of efficiency of locally administered antibiotics may also be explained by the route of administration and therefore the contact conditions between the microorganisms and the antibiotics used. In local treatments, concentrations can be up to 30 times higher than in systemic use, but the contact time is significantly shorter [28]. At these higher concentrations, local treatments need to exhibit bactericidal activity, making them similar to antiseptics. As demonstrated in our study, even though it is considered the first-line treatment for AV [28], clindamycin did not induce a significant log reduction in bacterial populations, including those of Gram-positive bacteria, under conditions simulating the likely contact times when used topically. At the same time, WHO and national agencies around the world believe that antibiotic resistance is accelerated by antibiotic misuse and overuse, and this may be consistent with our results concerning the two reference antibiotics evaluated, metronidazole and clindamycin.

This study constitutes the first in vitro demonstration of the current limits of local treatments based on the use of metronidazole or clindamycin, whether concerning the emergence of resistance or the intrinsic antibacterial properties of these antibiotics, given their spectrum of activity and the route of administration. Our results are consistent with published clinical data regarding the risks of lack of efficacy and disease recurrences associated with treatment with metronidazole or clindamycin. They are also consistent with the value of topical treatment with NPN, characterized by a broad antibacterial spectrum combined with a rapid bactericidal activity showing high clinical efficacy against infectious vaginitis. In clinical settings, this antibiotic/antifungal combination has shown high clinical efficacy against various types of infectious vaginitis, including bacterial infections (BV and AV), but also in vulvovaginal candidiasis and mixed infections [47,71]. To ensure a focused and clinically relevant evaluation, we limited this study to bacterial strains associated with bacterial vaginosis and aerobic vaginitis, even though NPN also contains nystatin, an antifungal agent particularly efficient against non-*albicans Candida* strains [72,73]. In 2015, Neut et al. highlighted the value of combining polymyxin B, neomycin, and antifungal compounds [74]. Their conclusion was based on the antimicrobial activity of these compounds, including their lack of interference with the in vitro growth of the *Lactobacillus* strains, but also on their pharmacological characteristics.

Over the years, antibiotic drug resistance has increased, whereas fewer new antibiotics have been identified [75]. In a recent study among women in the reproductive age group, nine of the sixty bacterial isolates (15%) were *S. aureus*, with seven being methicillin-resistant (MRSA) and five being clindamycin-resistant [76]. In another study, among 259 MRSA isolates from different body parts, only 8% were resistant to neomycin [77]. In this context, there is an increased interest in antibiotic combination therapy, as it could be associated with a decreased acquisition of drug resistance, synergistic effects, or drug rejuvenation [78]. The high and quick efficacy of NPN can therefore be explained by the additive and/or synergistic effect of the three active ingredients, in contrast to the comparators that consisted only of metronidazole or clindamycin. Actually, the neomycin-polymyxin B combination has been shown to present synergistic activity in vitro against Gram-negative and Gram-positive pathogens, including *S. aureus* [79]. Combination treatment can be of high value, in particular for the treatment of refractory or recurrent vaginal infection [80].

These results provide a basis for future clinical studies, taking into account local pharmacodynamic aspects and the likely bactericidal activity of the antibiotics evaluated. The originality of the work lies in the determination of bactericidal activity in the presence of a high protein load (simulating the vaginal fluid) during a short contact time, with initially high and rapidly decreasing doses of the antibiotics evaluated, mimicking the dynamic conditions of vaginal application, with initially high and rapidly decreasing antibiotic concentrations. This in vitro study, performed under conditions close to topical use in vaginal infections (in the presence of calf serum simulating the vaginal environment, after dilution, and for short periods of antibiotic contact with the tested bacterial strains) highlights the potential bactericidal activity of the NNP combination when locally administrated in comparison to metronidazole and clindamycin, respectively, against the main bacteria involved in BV and AV. Future studies may further characterize the synergistic activity of the NNP combination and its clinical implications, notably regarding the risk of resistance after treatment.

## 4. Materials and Methods

### 4.1. Antimicrobial Products

The Neomycin-Polymyxin B–Nystatin (NPN) antibiotic/antifungal combination was obtained from Polygynax^®^ vaginal capsules obtained from Laboratoire Innotech International (Arcueil, France) after cutting the envelope of the capsule using a sterile scalpel. These marketed vaginal capsules each contain 31.5–38.5 × 10^3^ IU of neomycin, 31.5–38.5 × 10^3^ IU of polymyxin B, and 90.0–110.0 × 10^3^ IU of nystatin. Metronidazole was extracted from Flagyl^®^ vaginal ovules (containing 500 mg of metronidazole per ovule), obtained from Sanofi-Aventis France, Gentilly, France. The contents of these ovules were extracted as described for NPN. Solutions were prepared by an initial 2-fold dilution (1/2 *v*/*v*) in Mueller–Hinton (MH) broth (Biomérieux, Craponne, France) or MH broth containing 2% (*w*/*v*) of Tween 80 (Sigma, Aldrich, St. Louis, MO, USA) for Polygynax^®^ and Flagyl^®^ contents, respectively. Further dilutions tested were prepared with the same media and ranged from 1/2 to 1/128 (*v*/*v*). No precipitate was noted.

As clindamycin vaginal capsules and cream were not available on the French market, the active ingredient, i.e., clindamycin hydrochloride, was purchased from Fischer Scientific (Illkirch, France). Assays were performed using a 20 mg/mL initial solution of clindamycin hydrochloride, corresponding to the described concentration in the vaginal cream (20 mg/g) after validation based on the determination of the minimal inhibitory concentrations (MICs) for the relevant strains using the microdilution method [81]. The dilutions tested were prepared as described for NPN and metronidazole.

The range of dilutions tested for each product was selected in order to assess the persistence of bactericidal activity in relation to pharmacodynamics at the vaginal level.

### 4.2. Bacterial Strains

The reference bacterial strains tested corresponded to the main Gram-positive and Gram-negative species implicated in BV and AV, respectively. They were obtained from the CIP (Pasteur Institute Collection, Paris, France) or from the DSM (German Collection of Microorganisms and Cell Cultures, Braunschweig, Germany). Strains selected for species involved in BV were of human origin, isolated from vaginal flora or the endometrium. Obligatory strains to be tested for the evaluation of bactericidal activity of antiseptics and disinfectants in the medical area (suspension test) were also selected (EN 13727 + A2 [76]) regarding AV. The bacterial strains and some of their characteristics, including resistance specificities and their subculture conditions, are presented in Table 1. The culture media were obtained from Biomérieux. Minimum inhibitory concentrations (MICs) were determined to ensure transparency. The results are presented in Supplementary Table S1. It is important to note that while these MIC values can indicate innate or acquired resistance, they are not relevant in view of the high antibiotic concentrations used in our experiments and the objective of defining rapid bactericidal activity. These concentrations were chosen to reflect typical clinical dosages used for local vaginal treatment. Specifically, the ranges used were 1060 mg/L to 16.5 mg/L for polymyxin B, 10,090 mg/L to 157.6 mg/L for neomycin, 10,000 mg/L to 156.25 mg/L for clindamycin, and approximately 125,000 mg/L to 1900 mg/L for metronidazole (based on a calculated maximal volume of 2 mL).

### 4.3. Assays

To determine the bactericidal activity of each dilution of the three antibiotic products evaluated, the experimental conditions specified in the EN 13727 + A2 standard [82] were adapted according to the indication of the tested products and the objectives. To simulate the vaginal microenvironment, antibiotic dilutions from 1/2 to 1/128 were prepared in MH broth including 10% sterile calf serum (Dutscher, ref. P30-3306, Bernolsheim, France).

Assays were performed at a temperature of 37 ± 1 °C under aerobiosis (AV) or anaerobiosis (BV) with contact durations of 1 h ± 30 s and 4 h ± 30 s. Briefly, a fresh suspension of each bacterial species was prepared in tryptone salt solution (Biomérieux) and added to 5 mL of the tested dilutions to obtain a bacterial count of approximately 10^8^ CFU/mL. At the end of each contact period, 1 mL of the mixture was transferred into the appropriate neutralizer (neutralizer 1: Tween 80 (10%), saponin (2%), lecithin (2%), and sodium thiosulfate (0.5%) (Sigma Aldrich, St. Louis, MO, USA)) in Trypticase Soy broth (Biomérieux) (neutralizer 2: Neutralizing Pharmacopeia Diluent (NDP) (Biomérieux)) (Table 1).

The absence of any toxic effects from the experimental conditions and the neutralizer, as well as the validation of the dilution and neutralization step, were checked as specified in the standard.

After neutralization and serial 10-fold dilutions, the CFU count of residual viable bacteria was determined in 1 mL of each dilution in duplicate. Calculations were performed in accordance with the EN standards on antiseptics and disinfectants. Briefly, the CFU values taken into account for the calculation were only those ≤330 CFU/plate. The CFU values (numeration in duplicate/dilution) obtained for one dilution or 2 successive dilutions were used to produce a weighted mean according to the following formula:

$$CFU/mL=\frac{c}{\left(n1+0.1n2\right)\times 10^{-x}}$$

where

- *c* is the sum of the CFU values taken into account;
- n1 is the number of CFU values taken into account for the lower dilution, i.e., 10^−x^;
- n2 is the number of CFU values taken into account for the higher dilution, i.e., 10^−(x+1)^;
- 10^−x^ is the dilution factor corresponding to the lower dilution.

For validation, regarding the results calculated by the weighted mean of two subsequent dilutions, the quotient of the mean of the two results should be no higher than 15 and no lower than 5.

The CFU counts determined for each product dilution, each strain, and each contact time, expressed in decimal logarithms (log), were subtracted from the baseline values prior to antibiotic exposure in order to calculate the log reductions in bacterial populations. According to EN 13727 + A2, the bactericidal effect was defined for conditions demonstrating at least a 5 decimal (log) reduction in the specified conditions. The bactericidal effect is considered as being fully achieved at 5-log, aligning with regulatory standards. This 5-log reduction corresponds to a reduction percentage of 99.999% (maximal value considered).

## 5. Conclusions

The present results support the locally effective rapid bactericidal activity of the NPN combination against a wide range of anaerobic and aerobic and Gram-positive and Gram-negative strains, whereas the antibiotics recommended by most guidelines (metronidazole for BV and clindamycin for AV) showed a low rapid bactericidal activity within the range of their expected local concentrations. These results correlate with the high clinical efficacy of the neomycin-polymyxin B-nystatin (NPN) combination against various types of infectious vaginitis, including bacterial vaginitis, bacterial vaginosis, and mixed vaginitis, with a fast reduction in symptoms [47]. Further studies exploring polymyxin B and neomycin interactions, especially in the context of topical use at high and low concentrations, could help to more thoroughly explore the mechanisms underlying their extended spectrum and rapid bactericidal activity. However, this study has the advantage of laying foundations for future work by explaining some of the shortcomings of current treatments and the value of alternative approaches, particularly in relation to topical administration.

## Figures and Tables

**Table 1:** Bacterial strains tested, main characteristics, and subculture conditions employed.

	Bacterial Strain	Subcultures	Main Characteristics
**Bacterial vaginosis**			
Gram-positive bacteria	*Atopobium (Fannyhessea) vaginae* DSM 15829 (T) *	Columbia agar + 5% sheep red cells, anaerobiosis ##	Type strain
	*Mobiluncus curtisii* DSM 23059 (T) *		Type strain
Gram-negativebacteria	*Prevotella bivia* CIP 105105T **	Columbia agar + 5% sheep red cells, anaerobiosis ##	Type strain
	*Gardnerella vaginalis* CIP 70.74T *		Type strain
**Aerobic vaginitis**			
Gram-positive bacteria	*Staphylococcus aureus* CIP 4.83 ***	Trypticase soy agar, aerobiosis #	*Recommended strain for microbiological assays of antibiotics* *Quality control strain for European and American Pharmacopeia*
	*Enterococcus hirae* CIP 58.55 ***	Trypticase soy agar, aerobiosis #	*Recommended strain for microbiological assays of antibiotics, assay of gramicidine, assays of tyrothricine, and assays of thiostreptone* *Quality control strain for European and American Pharmacopeia*
	*Enterococcus faecalis* CIP 103015T	Trypticase soy agar, aerobiosis #	Type strain
	*Streptococcus agalactiae* CIP 103227T	Trypticase soy agar, 5% CO_2_ #	Type strain Lancefield’s Group B Non-hemolytic
	*Streptococcus pyogenes* CIP 106884	Trypticase soy agar, aerobiosis #	Resistant to erythromycin, resistant to ciprofloxacin
	*Corynebacterium amycolatum* CIP 103452T	Columbia agar, aerobiosis #	Type strain
Gram-negative bacteria	*Pseudomonas aeruginosa* CIP 103467 ***	Trypticase soy agar, aerobiosis #	*Assay of slimicides* *Commercial germicides resistance*
	*Escherichia coli* CIP 54127 ***	Trypticase soy agar, aerobiosis #	H21 *Recommended strain for microbiological assays of antibiotics* *Quality control strain for European and American Pharmacopeia*
	*Proteus mirabilis* CIP 103181T	Trypticase soy agar, aerobiosis #	Type strain *Recommended strain for evaluation of antimicrobial preservatives*
	*Proteus hauseri* CIP 58.60	Trypticase soy agar, aerobiosis #	*Recommended strain for testing bactericides*
	*Klebsiella pneumoniae* CIP 82.91	Trypticase soy agar, aerobiosis #	Capsular type 3 *Recommended strain for assays of antimicrobial preservatives—bioresistance testing*
	*Klebsiella aerogenes* CIP 60.86T	Trypticase soy agar, aerobiosis #	*Recommended strain for assays of antimicrobial preservatives and slimicides*
	*Shigella flexneri* CIP 82.48T	Trypticase soy agar, aerobiosis #	Type strain Serotype 2a
	*Salmonella enterica enterica enteritidis* CIP 105150	Trypticase soy agar, aerobiosis #	Resistant to third-generation cephalosporins Resistant to cefoxitin
	*Yersinia enterocolitica* CIP 80.27T	Trypticase soy agar, aerobiosis #	Type strain Lysotype Xz Biotype 1B Serotype 7,8, Serotype 8, Serotype 19
	*Branhamella (Moraxella) catarrhalis* CIP 73.21T	Trypticase soy agar, aerobiosis #	Type strain
	*Haemophilus influenzae* CIP 102514T	Chocolate agar, 5% CO_2_ #	Type strain Presence of fimbriae but absence of the fimbrial genes ghfA, ghfD, and ghfE
	*Neisseria meningitidis A* CIP 73.10T	Chocolate agar, 5% CO_2_ #	Type strain *Reference strain for serogroup A*

T, type strain; * human vaginal strain; ** endometrium strain; *** EN 13727 + A2 obligatory strains; # neutralizer 1 (Polysorbate 80 (10%), Saponin (2%), Lecithin (2%), and sodium thiosulfate (0.5%)) completed to volume with Trypticase Soy broth; ## neutralizer 2 (NDP) (Biomérieux).

## Data Availability

The data presented in this study are available on request from the corresponding author.

## Supplementary Materials

The following supporting information can be downloaded at: https://www.mdpi.com/article/10.3390/ph18030340/s1. Supplementary materials: Minimum Inhibitory Concentration (MIC) Determination; Table S1: MIC determination of the used strains. Supplementary references [51,83].

## References

[1] Egan M.E., Lipsky M.S. (2000). Diagnosis of vaginitis. Am. Fam. Physician.

[2] Nyirjesy P., Sobel J.D. (2005). Advances in Diagnosing Vaginitis: Development of a New Algorithm. Curr. Infect. Dis. Rep..

[3] Sherrard J., Wilson J., Donders G., Mendling W., Jensen J.S. (2018). 2018 European (IUSTI/WHO) International Union against Sexually Transmitted Infections (IUSTI) World Health Organisation (WHO) Guideline on the Management of Vaginal Discharge. Int. J. STD AIDS.

[4] Bautista C.T., Wurapa E., Sateren W.B., Morris S., Hollingsworth B., Sanchez J.L. (2016). Bacterial Vaginosis: A Synthesis of the Literature on Etiology, Prevalence, Risk Factors, and Relationship with Chlamydia and Gonorrhea Infections. Mil. Med. Res..

[5] Coudray M.S., Madhivanan P. (2020). Bacterial Vaginosis—A Brief Synopsis of the Literature. Eur. J. Obstet. Gynecol. Reprod. Biol..

[6] Donders G.G.G., Vereecken A., Bosmans E., Dekeersmaecker A., Salembier G., Spitz B. (2002). Definition of a Type of Abnormal Vaginal Flora That Is Distinct from Bacterial Vaginosis: Aerobic Vaginitis. BJOG Int. J. Obstet. Gynaecol..

[7] Peebles K., Kiweewa F.M., Palanee-Phillips T., Chappell C., Singh D., Bunge K.E., Naidoo L., Makanani B., Jeenarain N., Reynolds D. (2021). Elevated Risk of Bacterial Vaginosis Among Users of the Copper Intrauterine Device: A Prospective Longitudinal Cohort Study. Clin. Infect. Dis..

[8] Jung H.-S., Ehlers M.M., Lombaard H., Redelinghuys M.J., Kock M.M. (2017). Etiology of Bacterial Vaginosis and Polymicrobial Biofilm Formation. Crit. Rev. Microbiol..

[9] Chen X., Lu Y., Chen T., Li R. (2021). The Female Vaginal Microbiome in Health and Bacterial Vaginosis. Front. Cell. Infect. Microbiol..

[10] Muzny C.A., Schwebke J.R. (2016). Pathogenesis of Bacterial Vaginosis: Discussion of Current Hypotheses. J. Infect. Dis..

[11] Muzny C.A., Taylor C.M., Swords W.E., Tamhane A., Chattopadhyay D., Cerca N., Schwebke J.R. (2019). An Updated Conceptual Model on the Pathogenesis of Bacterial Vaginosis. J. Infect. Dis..

[12] Schwebke J.R., Muzny C.A., Josey W.E. (2014). Role of *Gardnerella vaginalis* in the Pathogenesis of Bacterial Vaginosis: A Conceptual Model. J. Infect. Dis..

[13] Bradshaw C.S., Brotman R.M. (2015). Making Inroads into Improving Treatment of Bacterial Vaginosis—Striving for Long-Term Cure. BMC Infect. Dis..

[14] Fettweis J.M., Brooks J.P., Serrano M.G., Sheth N.U., Girerd P.H., Edwards D.J., Strauss J.F., Jefferson K.K., Buck G.A., The Vaginal Microbiome Consortium (2014). Differences in Vaginal Microbiome in African American Women versus Women of European Ancestry. Microbiology.

[15] Gilbert N.M., Lewis W.G., Li G., Sojka D.K., Lubin J.B., Lewis A.L. (2019). *Gardnerella vaginalis* and *Prevotella bivia* Trigger Distinct and Overlapping Phenotypes in a Mouse Model of Bacterial Vaginosis. J. Infect. Dis..

[16] Kroon S.J., Ravel J., Huston W.M. (2018). Cervicovaginal Microbiota, Women’s Health, and Reproductive Outcomes. Fertil. Steril..

[17] Leyva-Gómez G., Del Prado-Audelo M.L., Ortega-Peña S., Mendoza-Muñoz N., Urbán-Morlán Z., González-Torres M., González-Del Carmen M., Figueroa-González G., Reyes-Hernández O.D., Cortés H. (2019). Modifications in Vaginal Microbiota and Their Influence on Drug Release: Challenges and Opportunities. Pharmaceutics.

[18] Mendling W., Palmeira-de-Oliveira A., Biber S., Prasauskas V. (2019). An Update on the Role of Atopobium Vaginae in Bacterial Vaginosis: What to Consider When Choosing a Treatment? A Mini Review. Arch. Gynecol. Obstet..

[19] Palmeira-de-Oliveira R., Duarte P., Palmeira-de-Oliveira A., Das Neves J., Amaral M.H., Breitenfeld L., Martinez-de-Oliveira J. (2015). Women’s Experiences, Preferences and Perceptions Regarding Vaginal Products: Results from a Cross-Sectional Web-Based Survey in Portugal. Eur. J. Contracept. Reprod. Health Care.

[20] Palmeira-de-Oliveira R., Palmeira-de-Oliveira A., Martinez-de-Oliveira J. (2015). New Strategies for Local Treatment of Vaginal Infections. Adv. Drug Deliv. Rev..

[21] Srinivasan S., Hoffman N.G., Morgan M.T., Matsen F.A., Fiedler T.L., Hall R.W., Ross F.J., McCoy C.O., Bumgarner R., Marrazzo J.M. (2012). Bacterial Communities in Women with Bacterial Vaginosis: High Resolution Phylogenetic Analyses Reveal Relationships of Microbiota to Clinical Criteria. PLoS ONE.

[22] Rampersaud R., Randis T.M., Ratner A.J. (2012). Microbiota of the Upper and Lower Genital Tract. Semin. Fetal Neonatal Med..

[23] Turovskiy Y., Sutyak Noll K., Chikindas M.L. (2011). The Aetiology of Bacterial Vaginosis: Aetiology of Bacterial Vaginosis. J. Appl. Microbiol..

[24] Donders G.G.G., Bellen G., Grinceviciene S., Ruban K., Vieira-Baptista P. (2017). Aerobic Vaginitis: No Longer a Stranger. Res. Microbiol..

[25] Sangeetha T., Saroj G., Vasudha L. (2015). A Study of Aerobic Bacterial Pathogens Associated with Vaginitis in Reproductive Age Group Women (15–45 Years) and Their Sensitivity Pattern. Int. J. Res. Med. Sci..

[26] Tansarli G.S., Kostaras E.K., Athanasiou S., Falagas M.E. (2013). Prevalence and Treatment of Aerobic Vaginitis among Non-Pregnant Women: Evaluation of the Evidence for an Underestimated Clinical Entity. Eur. J. Clin. Microbiol. Infect. Dis..

[27] Tomusiak A., Heczko P.B., Janeczko J., Adamski P., Pilarczyk-Zurek M., Strus M. (2013). Bacterial Infections of the Lower Genital Tract in Fertile and Infertile Women from the Southeastern Poland. Ginekol. Pol..

[28] Vieira-Baptista P., Stockdale C.K., Sobel J. (2023). International Society for the Study of Vulvovaginal Disease Recommendations for the Diagnosis and Treatment of Vaginitis.

[29] Workowski K.A., Bachmann L.H., Chan P.A., Johnston C.M., Muzny C.A., Park I., Reno H., Zenilman J.M., Bolan G.A. (2021). Sexually Transmitted Infections Treatment Guidelines, 2021. MMWR Recomm. Rep..

[30] World Health Organization (2021). Guidelines for the Management of Symptomatic Sexually Transmitted Infections.

[31] Bagnall P., Rizzolo D. (2017). Bacterial Vaginosis: A Practical Review. J. Am. Acad. Physician Assist..

[32] Donders G.G.G., Zodzika J., Rezeberga D. (2014). Treatment of Bacterial Vaginosis: What We Have and What We Miss. Expert. Opin. Pharmacother..

[33] Laghi L., Picone G., Cruciani F., Brigidi P., Calanni F., Donders G., Capozzi F., Vitali B. (2014). Rifaximin Modulates the Vaginal Microbiome and Metabolome in Women Affected by Bacterial Vaginosis. Antimicrob. Agents Chemother..

[34] Schwebke J.R., Desmond R.A. (2007). A Randomized Trial of the Duration of Therapy with Metronidazole plus or Minus Azithromycin for Treatment of Symptomatic Bacterial Vaginosis. Clin. Infect. Dis..

[35] Bradshaw C.S., Sobel J.D. (2016). Current Treatment of Bacterial Vaginosis—Limitations and Need for Innovation. J. Infect. Dis..

[36] Machado D., Castro J., Palmeira-de-Oliveira A., Martinez-de-Oliveira J., Cerca N. (2016). Bacterial Vaginosis Biofilms: Challenges to Current Therapies and Emerging Solutions. Front. Microbiol..

[37] Machado A., Cerca N. (2015). Influence of Biofilm Formation by *Gardnerella vaginalis* and Other Anaerobes on Bacterial Vaginosis. J. Infect. Dis..

[38] Verstraelen H., Swidsinski A. (2019). The Biofilm in Bacterial Vaginosis: Implications for Epidemiology, Diagnosis and Treatment: 2018 Update. Curr. Opin. Infect. Dis..

[39] Bilardi J., Walker S., McNair R., Mooney-Somers J., Temple-Smith M., Bellhouse C., Fairley C., Chen M., Bradshaw C. (2016). Women’s Management of Recurrent Bacterial Vaginosis and Experiences of Clinical Care: A Qualitative Study. PLoS ONE.

[40] Bradshaw C.S., Morton A.N., Hocking J., Garland S.M., Morris M.B., Moss L.M., Horvath L.B., Kuzevska I., Fairley C.K. (2006). High Recurrence Rates of Bacterial Vaginosis over the Course of 12 Months after Oral Metronidazole Therapy and Factors Associated with Recurrence. J. Infect. Dis..

[41] Vodstrcil L.A., Muzny C.A., Plummer E.L., Sobel J.D., Bradshaw C.S. (2021). Bacterial Vaginosis: Drivers of Recurrence and Challenges and Opportunities in Partner Treatment. BMC Med..

[42] Hay P. (2009). Recurrent Bacterial Vaginosis. Curr. Opin. Infect. Dis..

[43] Fan A., Yue Y., Geng N., Zhang H., Wang Y., Xue F. (2013). Aerobic Vaginitis and Mixed Infections: Comparison of Clinical and Laboratory Findings. Arch. Gynecol. Obstet..

[44] Abou Chacra L., Fenollar F., Diop K. (2022). Bacterial Vaginosis: What Do We Currently Know?. Front. Cell. Infect. Microbiol..

[45] Bohbot J.-M., Sednaoui P., Verriere F., Achhammer I. (2012). Diversité étiologique des vaginites. Gynécol. Obs. Fertil..

[46] Qian Z., Zhu H., Zhao D., Yang P., Gao F., Lu C., Yin Y., Kan S., Chen D. (2021). Probiotic *Lactobacillus* sp. Strains Inhibit Growth, Adhesion, Biofilm Formation, and Gene Expression of Bacterial Vaginosis-Inducing *Gardnerella vaginalis*. Microorganisms.

[47] Bohbot J.M., Goubard A., Aubin F., Mas Y., Coatantiec E., Lucas N., Verrière F. (2019). PRISM Study: Comparison of a Nystatin-Neomycin-Polymyxin B Combination with Miconazole for the Empirical Treatment of Infectious Vaginitis. Méd. Mal. Infect..

[48] Velkov T., Roberts K.D., Nation R.L., Thompson P.E., Li J. (2013). Pharmacology of Polymyxins: New Insights into an ‘Old’ Class of Antibiotics. Future Microbiol..

[49] Trimble M.J., Mlynárčik P., Kolář M., Hancock R.E.W. (2016). Polymyxin: Alternative Mechanisms of Action and Resistance. Cold Spring Harb. Perspect. Med..

[50] Lima M.R., Ferreira G.F., Nunes Neto W.R., Monteiro J.d.M., Santos Á.R.C., Tavares P.B., Denadai Â.M.L., Bomfim M.R.Q., dos Santos V.L., Marques S.G. (2019). Evaluation of the Interaction between Polymyxin B and *Pseudomonas aeruginosa* Biofilm and Planktonic Cells: Reactive Oxygen Species Induction and Zeta Potential. BMC Microbiol..

[51] Falagas M.E., Kasiakou S.K., Saravolatz L.D. (2005). Colistin: The Revival of Polymyxins for the Management of Multidrug-Resistant Gram-Negative Bacterial Infections. Clin. Infect. Dis..

[52] Nation R.L., Velkov T., Li J. (2014). Colistin and Polymyxin B: Peas in a Pod, or Chalk and Cheese?. Clin. Infect. Dis..

[53] Davies J., Davis B.D. (1968). Misreading of Ribonucleic Acid Code Words Induced by Aminoglycoside Antibiotics: The Effect of Drug Concentration. J. Biol. Chem..

[54] Feldman M.B., Terry D.S., Altman R.B., Blanchard S.C. (2010). Aminoglycoside Activity Observed on Single Pre-Translocation Ribosome Complexes. Nat. Chem. Biol..

[55] Belardinelli R., Sharma H., Peske F., Rodnina M.V. (2021). Perturbation of Ribosomal Subunit Dynamics by Inhibitors of tRNA Translocation. RNA.

[56] Misumi M., Nishimura T., Komai T., Tanaka N. (1978). Interaction of Kanamycin and Related Antibiotics with the Large Subunit of Ribosomes and the Inhibition of Translocation. Biochem. Biophys. Res. Commun..

[57] Cabañas M.J., Vázquez D., Modolell J. (1978). Inhibition of Ribosomal Translocation by Aminoglycoside Antibiotics. Biochem. Biophys. Res. Commun..

[58] Deng Z.-L., Gottschick C., Bhuju S., Masur C., Abels C., Wagner-Döbler I. (2018). Metatranscriptome Analysis of the Vaginal Microbiota Reveals Potential Mechanisms for Protection against Metronidazole in Bacterial Vaginosis. Msphere.

[59] Ferris M.J., Masztal A., Martin D.H. (2004). Use of Species-Directed 16S rRNA Gene PCR Primers for Detection of Atopobium Vaginae in Patients with Bacterial Vaginosis. J. Clin. Microbiol..

[60] Gal M., Brazier J.S. (2004). Metronidazole Resistance in *Bacteroides* spp. Carrying *Nim* Genes and the Selection of Slow-Growing Metronidazole-Resistant Mutants. J. Antimicrob. Chemother..

[61] Ravel J., Brotman R.M., Gajer P., Ma B., Nandy M., Fadrosh D.W., Sakamoto J., Koenig S.S., Fu L., Zhou X. (2013). Daily Temporal Dynamics of Vaginal Microbiota before, during and after Episodes of Bacterial Vaginosis. Microbiome.

[62] Schuyler J.A., Mordechai E., Adelson M.E., Sobel J.D., Gygax S.E., Hilbert D.W. (2016). Identification of Intrinsically Metronidazole-Resistant Clades of *Gardnerella vaginalis*. Diagn. Microbiol. Infect. Dis..

[63] Sobel J.D. (2021). Recurrent Bacterial Vaginosis, Relapse or Reinfection: The Role of Sexual Transmission. BJOG.

[64] Lee C.Y., Cheu R.K., Lemke M.M., Gustin A.T., France M.T., Hampel B., Thurman A.R., Doncel G.F., Ravel J., Klatt N.R. (2020). Quantitative Modeling Predicts Mechanistic Links between Pre-Treatment Microbiome Composition and Metronidazole Efficacy in Bacterial Vaginosis. Nat. Commun..

[65] Alves P., Castro J., Sousa C., Cereija T.B., Cerca N. (2014). *Gardnerella vaginalis* Outcompetes 29 Other Bacterial Species Isolated from Patients with Bacterial Vaginosis, Using in an in Vitro Biofilm Formation Model. J. Infect. Dis..

[66] Castro J., Alves P., Sousa C., Cereija T., França Â., Jefferson K.K., Cerca N. (2015). Using an In-Vitro Biofilm Model to Assess the Virulence Potential of Bacterial Vaginosis or Non-Bacterial Vaginosis *Gardnerella vaginalis* Isolates. Sci. Rep..

[67] Janulaitiene M., Gegzna V., Baranauskiene L., Bulavaitė A., Simanavicius M., Pleckaityte M. (2018). Phenotypic Characterization of *Gardnerella vaginalis* Subgroups Suggests Differences in Their Virulence Potential. PLoS ONE.

[68] Li T., Zhang Z., Wang F., He Y., Zong X., Bai H., Liu Z. (2020). Antimicrobial Susceptibility Testing of Metronidazole and Clindamycin against *Gardnerella vaginalis* in Planktonic and Biofilm Formation. Can. J. Infect. Dis. Med. Microbiol..

[69] Swidsinski A., Mendling W., Loening-Baucke V., Swidsinski S., Dörffel Y., Scholze J., Lochs H., Verstraelen H. (2008). An Adherent *Gardnerella vaginalis* Biofilm Persists on the Vaginal Epithelium after Standard Therapy with Oral Metronidazole. Am. J. Obstet. Gynecol..

[70] Mollin A., Katta M., Sobel J.D., Akins R.A. (2022). Association of Key Species of Vaginal Bacteria of Recurrent Bacterial Vaginosis Patients before and after Oral Metronidazole Therapy with Short- and Long-Term Clinical Outcomes. PLoS ONE.

[71] Bohbot J.-M., Sednaoui P., Verriere F. (2014). Nystatin-Neomycin-Polymyxin B Combination: Efficacy and Tolerance as 1st-Line Local Treatment of Infectious Vaginitis. Open J. Obstet. Gynecol..

[72] Choukri F., Benderdouche M., Sednaoui P. (2014). In Vitro Susceptibility Profile of 200 Recent Clinical Isolates of *Candida* spp. to Topical Antifungal Treatments of Vulvovaginal Candidiasis, the Imidazoles and Nystatin Agents. J. Mycol. Médicale.

[73] Wang F.-J., Zhang D., Liu Z.-H., Wu W.-X., Bai H.-H., Dong H.-Y. (2016). Species Distribution and In Vitro Antifungal Susceptibility of Vulvovaginal *Candida* Isolates in China. Chin. Med. J..

[74] Neut C., Verrière F., Nelis H.J., Coenye T. (2015). Topical Treatment of Infectious Vaginitis: Effects of Antibiotic, Antifungal and Antiseptic Drugs on the Growth of Normal Vaginal *Lactobacillus* Strains. Open J. Obstet. Gynecol..

[75] Hutchings M.I., Truman A.W., Wilkinson B. (2019). Antibiotics: Past, Present and Future. Curr. Opin. Microbiol..

[76] Namitha B.N., Natarajan A. (2023). Vulvovaginitis Due to Methicillin-Resistant and Methicillin-Sensitive *Staphylococcus aureus* among Women in Reproductive Age-Group. J. Clin. Sci. Res..

[77] Suzuki M., Yamada K., Nagao M., Aoki E., Matsumoto M., Hirayama T., Yamamoto H., Hiramatsu R., Ichiyama S., Iinuma Y. (2011). Antimicrobial Ointments and Methicillin-Resistant *Staphylococcus aureus* USA300. Emerg. Infect. Dis..

[78] Coates A.R.M., Hu Y., Holt J., Yeh P. (2020). Antibiotic Combination Therapy against Resistant Bacterial Infections: Synergy, Rejuvenation and Resistance Reduction. Expert Rev. Anti-Infect. Ther..

[79] Tempera G., Mangiafico A., Genovese C., Giudice E., Mastrojeni S., Nicolosi D., Furneri P.M. (2009). In Vitro Evaluation of the Synergistic Activity of Neomycin-Polymyxin B Association against Pathogens Responsible for Otitis Externa. Int. J. Immunopathol. Pharmacol..

[80] Muzny C.A., Sobel J.D. (2022). The Role of Antimicrobial Resistance in Refractory and Recurrent Bacterial Vaginosis and Current Recommendations for Treatment. Antibiotics.

[81] Giske C.G., Turnidge J., Cantón R., Kahlmeter G. (2022). Update from the European Committee on Antimicrobial Susceptibility Testing (EUCAST). J. Clin. Microbiol..

[82] (2015). Antiseptiques et Désinfectants Chimiques—Essai Quantitatif de Suspension Pour L’évaluation de L’activité Bactéricide en Médecine—Méthode D’essai et Prescriptions (Phase 2, Étape 1).

[83] Poirel L., Jayol A., Nordmann P. (2017). Polymyxins: Antibacterial Activity, Susceptibility Testing, and Resistance Mechanisms Encoded by Plasmids or Chromosomes. Clin. Microbiol. Rev..

