# Supplementary material for: In Vitro Bactericidal Activity of a Neomycin-Polymyxin B-Nystatin Combination Compared to Metronidazole and Clindamycin Against the Main Bacteria Involved in Bacterial Vaginosis and Aerobic Vaginitis
Source: Pharmaceuticals (Basel). 2025 Feb 27;18(3):340. doi: 10.3390/ph18030340 (PMC11946053; doi:10.3390/ph18030340)
Supplement: Supplementary file 1 [file pharmaceuticals-18-00340-s001.zip › pharmaceuticals-3280841-supplementary.pdf]

Supplementary materials

Minimum Inhibitory Concentration (MIC) Determination

MIC values were determined using the broth microdilution method following EUCAST and CA-SFM guidelines, with the exception of the use of Mueller-Hinton Broth supplemented with 10% fetal calf serum (FCS) for the growth of anaerobic bacteria instead of Brucella Broth. The antimicrobial agents tested included neomycin, polymyxin B, clindamycin, and metronidazole, each assessed separately using serial twofold dilutions.

Bacterial suspensions were prepared to a final inoculum of 5×10^5 CFU/mL, and microplates were incubated at 35 ± 2°C for 18–24 hours (aerobes/facultative anaerobes) or 48 hours (anaerobes) under appropriate atmospheric conditions. MIC values were defined as the lowest concentration of the antimicrobial combination that resulted in the complete inhibition of visible bacterial growth. Assays were performed in duplicate. Only results giving identical MIC values between the 2 tests or with a dilution difference are considered valid. In the latter case, the 2 detected values are taken into account and mentioned in the results table.

Quality control was ensured using reference strains recommended by EUCAST (e.g., *Escherichia coli* ATCC 25922, *Staphylococcus aureus* CIP 103429, *Streptococcus pneumoniae* CIP 104340 and *Bacteroides thetaiotaomicron* ATCC 29741) for MIC validation.

Results were interpreted based on the established European Committee on Antimicrobial Susceptibility Testing EUCAST V15.0 2025 breakpoints for topical agents when available. If unavailable, EUCAST V15.0 2025 breakpoints for systemic use were applied. In other cases, breakpoints from the Antibiogram Committee of the French Society of Microbiology (Comité de l'antibiogramme de la Société Française de Microbiologie, CA-SFM) V1.0 2024 and the Clinical and Laboratory Standards Institute (CLSI) M100-2023 were used. If no natural resistance or breakpoints were described, literature was searched. For Polymyxin two references supporting resistance patterns were retrieved (Falagas and Kasiakou, 2005; Poirel et al., 2017).

References

Falagas, M.E., Kasiakou, S.K., 2005. Colistin: the revival of polymyxins for the management of multidrug-resistant gram-negative bacterial infections. Clin Infect Dis 40, 1333–1341. https://doi.org/10.1086/429323

Poirel, L., Jayol, A., Nordmann, P., 2017. Polymyxins: Antibacterial Activity, Susceptibility Testing, and Resistance Mechanisms Encoded by Plasmids or Chromosomes. Clin Microbiol Rev 30, 557–596. https://doi.org/10.1128/CMR.00064-16

Supplementary Table 1: MIC determination of the used strains

		Neomycin		Polymyxin B		Clindamycin		Metronidazole	
**Bacterial strain**	**CMI media**	**MIC (mg/L)**	**Interpretation**	**MIC (mg/L)**	**Interpretation**	**MIC (mg/L)**	**Interpretation**	**MIC (mg/L)**	**Interpretation**
**Bacterial Vaginosis – Gram-positive bacteria**									
Atopobium vaginae DSM 15829	Mueller-Hinton Broth + 10% fetal calf serum<br>36°C anaerobiosis Incubation 48h	125	R[3]	> 500	R[3]#			125	R*[4]
Mobilincus curtisii DSM 23059		0,98	R[3]	7,8	R[3]#			63	R
**Bacterial Vaginosis – Gram-negative bacteria**									
Gardnerella vaginalis CIP 7074T	Mueller-Hinton Broth + 10% fetal calf serum<br>36°C anaerobiosis Incubation 48h	125	ND	> 500	ND			15,6	S[4]
Prevotella bivia CIP 105105T		125	R[3]	31,25 - 63	R#			1,95	S[4]
**Aerobic Vaginitis – Gram-positive bacteria**									
***S. aureus* CIP 4.83**	Mueller-Hinton Broth<br>36°C aerobiosis Incubation 24h	0,98	S[1]	63	R[3]	0,06	S[4]		
***E. hirae* CIP 58.55**		31,25	R[2]	500	R[2]	15,63	R[2]		
***E. faecalis* CIP 103015T**		31,25 - 63	R[2]	500	R[2]	1,95	R[3]		
***S. agalactiae* CIP 103227T**		15,6	R[3]	31,25	R[3]	31,25	R*[2]		
***S. pyogenes* CIP 106884**		7,8	R[3]	15,6	R[3]	250	R*[2]		
***C. amycolatum* CIP 107291T**		0,12	ND	1,95	R[3]	0,06	S[2]		
**Aerobic Vaginitis – Gram-negative bacteria**									
***P. aeruginosa* CIP 103467**	Mueller-Hinton Broth<br>36°C aerobiosis Incubation 24h	3,9	R[3]	1,95	S[1]	≥500	R[2]		
***E. coli* CIP 54.127**		1,95	S[1]	1,95	S[1]	15,6	R[3]		
***P. mirabilis* CIP 103181T**		7,8	S[1]	> 500	R[3]	500	R[3]		
***P. hauserii* CIP 58.60**		1,95	S[1]	15,63	R[3]	63	R[3]		
***K. pneumoniae* CIP 82.91**		0,98 – 1,95	S[1]	7,8	R*[1]	125	R[3]		
***K. aerogenes* CIP 60.86T**		1,95	S[1]	0,98 – 1,95	S[1]	125	R[3]		

***S. flexneri* CIP 82.48T**		3,9	S[1]	0,98	S[1]	63	R[3]		
***S. enterica entirica enteritidis* CIP 105150**		1,95	S[1]	1,95	S[1]	125 - 250	R[3]		
***Y. enterocolitica* CIP 80.27T**		1,95	S[1]	0,49	S[1]	7,8	R[3]		
***B. catarrhalis* CIP 73.21T**		0,49	ND	1,95	ND	0,49	R[3]		
***H. influenzae* CIP 102514T**	Mueller-Hinton Broth + 5 % laked horse blood + 20 mg / L β-NAD (MH-F) 36°C 5% CO2 Incubation 24h	3,9	ND	0,24	ND	3,9	R[3]		
***Neisseria meningitidis A* CIP 73.10T**		3,9	ND	31,25	R[3]	1,95	R[3]		

R: natural resistance; R*: MIC value above the described breakpoint ; S: susceptible strain according to defined breakpoints; ND: no breakpoint described or no indication for systemic treatment; according to [1]EUCAST V15.0 - topical agent; [2]EUCAST V15.0 - systemic use; [3]CA-SFM 2024; [4]CLSI M100 2023. #confirmed in the literature by Falgas *et al*., 2005 and Poirel *et al*., 2017.
